# The prevalence and antimicrobial resistance of respiratory pathogens isolated from feedlot cattle in Canada

**DOI:** 10.3389/fmicb.2025.1497402

**Published:** 2025-01-28

**Authors:** Porjai Rattanapanadda, Dana Ramsay, Alyssa Butters, Calvin W. Booker, Sherry J. Hannon, Steve Hendrick, Joyce Van Donkersgoed, Brian N. Warr, Sheryl P. Gow, Paul S. Morley

**Affiliations:** ^1^Department of Veterinary Medicine, College of Veterinary Medicine, National Chung Hsing University, Taichung, Taiwan; ^2^Department of Livestock Development, Ministry of Agriculture and Cooperatives, Bangkok, Thailand; ^3^Department of Large Animal Clinical Sciences, University of Saskatchewan, Saskatoon, SK, Canada; ^4^Faculty of Veterinary Medicine, University of Calgary, Calgary, AB, Canada; ^5^Telus Agriculture, Okotoks, AB, Canada; ^6^Coaldale Veterinary Clinic, Lethbridge, AB, Canada; ^7^Dr. Joyce Van Donkersgoed Inc., Coaldale, AB, Canada; ^8^Veterinary Agri Health Services, Rocky View, AB, Canada; ^9^Canadian Integrated Program for Antimicrobial Resistance Surveillance, Public Health Agency of Canada, Saskatoon, SK, Canada; ^10^Veterinary Education, Research and Outreach (VERO) Program, College of Veterinary Medicine and Biomedical Sciences, Texas A&M University, Canyon, TX, United States

**Keywords:** bovine respiratory disease, antimicrobial resistance, *Mannheimia haemolytica*, *Pasteurella multocida*, *Histophilus somni*

## Abstract

**Objectives:**

The purpose of this study was to characterize the prevalence of antimicrobial resistance in *Mannheimia haemolytica*, *Pasteurella multocida*, and *Histophilus somni* isolated from healthy feedlot cattle over 2 years, and investigate factors potentially associated with recovery of resistant isolates.

**Methods:**

Deep-guarded nasopharyngeal (NP) swabs were used to sample feedlot cattle in multiple randomly selected feedlots (2019 *n* = 21, 2020 *n* = 26) at 2 timepoints. NP swabs were collected from 16 animals in each enrolled group upon entry processing and later in the feeding period. Cattle from the same groups (not necessarily the same animals) were sampled at both timepoints. Susceptibility testing was performed using the broth microdilution.

**Results:**

A total of 1,392 cattle within 47 housing groups were sampled over 2 years, providing 625 bacterial isolates for investigation. *Pasteurella multocida* (27.4%) was the most frequently isolated BRD organism, followed by *H. somni* (9%) and *M. haemolytica* (8.5%). Resistance to ≥3 antimicrobial classes was detected in 2.4% of *M. haemolytica*, 3.4% of *H. somni*, and 21.3% of *P. multocida* isolates. Potential associations were investigated between recovery of resistant organisms and time of year at sampling (quarter), sampling timepoint (arrival or second sample), days on feed (DOF) at sampling, animal age categories, and BRD risk categories. There was a significant (*p <* 0.05) increase in resistance prevalence after arrival for macrolide drugs in *M. haemolytica*, and for ampicillin, danofloxacin, enrofloxacin, spectinomycin, gamithromycin, tildipirosin, tulathromycin and tetracycline in *P. multocida* isolates. Resistance was higher in calves than in yearlings for tulathromycin in *H. somni*, and for gamithromycin, spectinomycin, tulathromycin, tildipirosin, and tetracycline for *P. multocida* (*p* < 0.05) Resistance to tetracycline, tildipirosin, and tulathromycin decreased between 61–80 DOF and 81–100 DOF when compared to 20–40 DOF, whereas for spectinomycin, resistance was lower in cattle sampled between 61–80 DOF than those sampled at 20–40 DOF for *P. multocida*.

**Discussion:**

The diversity of AMR profiles and associated risk factors between the BRD pathogens studied, underscores the importance of including all three organisms in future AMR studies in beef cattle.

## 1 Introduction

Respiratory disease is one of the costliest diseases in North American feedlot cattle, is one of the leading causes of morbidity and mortality in the fed cattle industry worldwide, and is the most common reason for treating beef cattle with injectable antimicrobial drugs (Murray et al., 2016b; Peel, 2020). Bovine respiratory disease complex (BRD) involves various pathogens, including viruses, mycoplasma, and bacteria. *Mannheimia haemolytica*, *Pasteurella multocida*, and *Histophilus somni* are typically considered the three major bacterial pathogens contributing to BRD. Economic losses stem from several factors including reductions in daily weight gain, reduced feed efficiency, weight loss, impact on beef quality, losses from animal death, increased labor costs to manage affected animals, and the cost of metaphylactic or therapeutic antimicrobial use (AMU) (Fernandez et al., 2020; Griffin et al., 2010). Peel (2020) estimated that the average cost of BRD treatment per infected animal was $23.60 USD and that over $75 million USD is spent on BRD treatments annually in the United States. Additionally, antimicrobial resistant BRD pathogens can lead to treatment failure and further escalate the losses attributable to BRD (Booker and Lubbers, 2020). The substantial cost of BRD-associated morbidity and mortality underscores the importance of the prevention and control of BRD in feedlot management.

*Mannheimia haemolytica*, *P. multocida*, and *H. somni* are pathobionts and are often considered normal constituents of microbial communities in the upper respiratory tract of cattle (Erickson et al., 2017; Schonecker et al., 2020). It is generally believed that stress, environmental conditions, and viral co-infections of the host can lead to inflammation and immunosuppression, allowing these bacteria to proliferate opportunistically in the respiratory tract. Metaphylaxis with antimicrobial drugs (AMDs) and vaccination are frequently used to prevent or control these infections (Erickson et al., 2017, Schonecker et al., 2020), albeit with varying success (O'Connor et al., 2019a; O'Connor et al., 2019b).

Metaphylactic AMU has become one of the most important methods for preventing and managing BRD in cattle and improving overall animal health (Ives and Richeson, 2015; O'Connor et al., 2019b). Despite the importance of metaphylaxis in managing BRD the use of AMDs in the beef industry may introduce pressure for the selection of antimicrobial-resistant (AMR) bacteria (Cameron and McAllister, 2016; Crosby et al., 2023; Erickson et al., 2017; Portis et al., 2012).

Monitoring trends in AMR supports the development of mitigation strategies for controlling AMR and facilitates a greater understanding of the risk factors associated with AMR in BRD pathogens (Murray et al., 2016a). This study aimed to describe the prevalence of AMR in *M. haemolytica*, *P. multocida,* and *H. somni* recovered from a broad sample of healthy feedlot cattle and evaluate potential risk factors associated with recovery of isolates resistant to AMD’s.

## 2 Materials and methods

### 2.1 General study overview

Nasopharyngeal (NP) swab samples were collected in 2019 and 2020 from healthy feedlot cattle at multiple randomly selected Canadian beef feedlots as part of a surveillance effort examining AMR in BRD pathogens (*M. haemolyica*, *P. multocida,* and *H. somni*). Once annually, 16 individual cattle were sampled at each participating feedlot at the time of entry processing (arrival timepoint). The same group of cattle, but not necessarily the same cattle, were sampled again when handled later in the feeding period (rehandling timepoint). After recovery of pathogen isolates, antimicrobial susceptibility testing was conducted using the microbroth dilution method. Factors potentially associated with AMR were examined, including sample year, sample quarter, sampling timepoint, animal age, animal weight, BRD risk category, and days on feed (DOF). All animal handling protocols were reviewed and approved by an Animal Care Committee before conducting this research (protocol number FHMS-19031). Inclusion criteria for the surveillance program were developed to allow enrollment of eligible feedlots in proportion to feedlot capacity and the number of fed cattle in the target provinces of Alberta, Saskatchewan, and Ontario, where 90% of feedlots are located.

### 2.2 Study population

The inclusion criteria require that enrolled feedlots have a one-time capacity of >1,000 animals, have cattle going directly to slaughter on-site, and have an established veterinarian-client-patient relationship with the veterinarian that enrolls the feedlot. From an anonymized list of feedlots provided by participating veterinarians, eligible feedlots were stratified by size (1,000–5,000 cattle, 5,001–10,000 cattle, 10,001–20,000 cattle, and > 20,000 cattle) and province and then randomly chosen in proportion to that type of feedlot’s contribution to Canada’s overall fed cattle production (See details in Supplementary methods). The final sample was comprised of 21 feedlots in 2019 and 26 feedlots in 2020. Selection was intended to reflect the diversity of cattle sources and production methods among the majority of feedlot cattle produced in Canada.

A single group of cattle assigned to be managed together, at least until the second sampling timepoint, was enrolled for sampling at each feedlot annually. Convenience sampling was used to select each group and the individual cattle at each feedlot. The veterinary practice that enrolled the feedlot was responsible for sample collection. Samples were to be shipped to the laboratory by mid-week to ensure timely processing, therefore, the group of cattle that were sampled depended the laboratory timelines, the schedule of the veterinarian and the availability of arrival cattle at the feedlot,

### 2.3 Sample collection

Sampling occurred at feedlot entry and later when cattle were rehandled for routine management procedures. Sixteen individual animals from each enrolled group were sampled at arrival processing, and 16 individual animals (not necessarily the same animals) from the same enrolled group were sampled at rehandling. The timing for the collection of samples at rehandling was variable across feedlots.

Nasopharyngeal (NP) samples were collected using sterile double-guarded swabs (Sterile Equine Double Guarded Uterine Swab, VetSource Canada Inc., Cambridge, Canada) using a standardized procedure established by the study. The sample collector wore exam gloves that were changed between each animal. Cattle were appropriately restrained in a squeeze chute, and the swabs were passed through the ventral meatus to the pharynx (approximately 10 to 14 inches, depending on the size of the animal), advanced through the guard to collect samples, retracted into the guarded sheath and removed from the nasal passage of the animal. Swab tips were aseptically placed in tubes containing Amies media (Micronostyx, Ottawa, Canada), placed in a cooler, and shipped on ice to Prairie Diagnostic Services (PDS) in Saskatoon, SK, Canada, for isolation of BRD pathogens and susceptibility testing.

### 2.4 Bacterial culture and species confirmation

NP swabs were plated on blood and chocolate agar plates (Oxoid, Nepean, ON, Canada) and incubated at 35 ± 2°C for 18–24 h in an environment containing 5% CO_2_ for isolation of *M. haemolytica*, *P. multocida,* and *H. somni*. Suspected colonies of pathogens were selected based on phenotypic characteristics such as the production of *β*-hemolysis (*M. haemolytica*), mucoid colonies (*P. multocida*), or yellow pigment (*H. somni*) at 18–24 h of incubation. After 48 h of incubation, plates were examined again with specific emphasis on identifying new *H. somni* growth. Species identification of suspect colonies was evaluated using the Matrix Assisted Laser Desorption and Ionization Time of Flight (MALDI-TOF) Mass Spectrometry System (Bruker Daltonics Ltd. East Milton, ON, Canada) according to the manufacturer’s operating standards and procedures. One isolate of each organism of interest with confirmed identification was stored per sample in Tryptic Soy Broth (TSB) containing 15% glycerol at −80°C until processed for susceptibility testing.

### 2.5 Antimicrobial susceptibility testing and breakpoint interpretation

The antimicrobial susceptibility of *M. haemolytica, P. multocida*, and *H. somni* isolates was determined by broth microdilution on a commercially available bovine panel (BOPO7F; Sensitire; Trek Diagnostic Systems, Cleveland, OH, USA), using Clinical Laboratory Standards Institute (CLSI) Guidelines and standards (CLSI, 2020) for testing and quality control. Control strains used as references in these analyses were *M. haemolytica* ATCC 33396, *P. multocida* ATCC 12945, and *H. somni* ATCC 700025, respectively. Thawed *M. haemolytica* and *P. multocida* isolates were regrown on blood agar plates at 37°C for 18–24 h, while *H. somni* isolates were grown on chocolate agar plates at 37°C with 5% CO_2_ for 20–24 h. For each sample and organism, a bacterial suspension reaching a final concentration of McFarland 0.5 was prepared, inoculated onto the BOPO7F plates, and incubated at 37°C with 5% CO_2_ for 20–24 h for *H. somni* or 37°C for 18–24 h for *M. haemolytica* and *P. multocida*, respectively.

The minimum inhibitory concentration (MIC) for each drug and isolate was interpreted with reference to the antimicrobial susceptibility breakpoints established by CLSI (2020); isolates were assigned to one of the susceptible, intermediate, or resistant categories on this basis (Supplementary Table S1). For the purposes of analysis and to permit a dichotomous outcome, “intermediate” isolates were categorized as “not resistant” to the respective AMD. CLSI breakpoints for the organisms of interest were not available for clindamycin, gentamicin, neomycin, tiamulin, trimethoprim-sulfamethoxazole, tylosin tartrate, or sulfadimethoxine, and only MIC data were summarized. The MIC_50_ and MIC_90_ (minimum concentrations at which 50 and 90% of isolates are inhibited from growing, respectively), MIC range, percentage of resistance, and multiple drug resistance (resistance to ≥3 AMD classes) were summarized for each bacterial species.

### 2.6 Statistical analysis

The prevalence of resistance among isolates was categorized for each species and AMD: rare (<0.1%), very low (0.1 to 1.0%), low (>1.0 to 10.0%), moderate (>10.0 to 20.0%), high (>20.0 to 50.0%), very high (>50.0 to 70.0%) and extremely high (>70.0%) (European Food Safety Authority European Centre For Disease Prevention Control, 2021). AMDs were classified relative to their importance to human medicine as outlined by the Veterinary Drugs Directorate, Health Canada (Health Canada, 2009). Briefly, AMDs are classified as Very Highly Important in Human Medicine (Category I) when essential for treating serious bacterial infections, and there is no or limited availability of alternative antimicrobials for effective treatment. Antimicrobials classified in Categories II through IV are of decreasing importance to human medicine, with the latter including those that are not currently used to treat infections in humans.

The susceptibility data were exported from a central repository to Stata/IC 14.2 2 (StataCorp LLC, College Station, TX, USA) for statistical analyses, and population-averaged prevalences of resistance to AMD with CLSI breakpoints were calculated to account for the hierarchical population structures (clustering) of isolates obtained from animals within different pens and feedlots. Null binomial response models were fitted using generalized estimating equations (xtgee) with a binary outcome, logit-link function, and exchangeable correlation structures to provide adjusted prevalence estimates with 95% confidence intervals. Separate models were used in estimating resistance prevalence for each of the antimicrobial drugs that were tested. If the GEE model did not converge, the standard error for the raw proportion was adjusted to account for clustering by feedlot. If the prevalence of resistance was 0%, a confidence interval calculator was used to estimate an exact upper confidence interval only (StataCorp LLC, College Station, TX, USA).

UpSet plots were produced to visualize intersections between the number of isolates with unique AMR patterns for the different respiratory pathogens (*M. haemolytica*, *P. multocida, H. somni*) at arrival and rehandling timepoints, by animal age class (calf vs. yearling) and BRD risk status (high vs. low). These plots were created in RStudio (R Core Team, 2023; Posit Team, 2023) using the ComplexUpset (Krassowski et al., 2022; Lex et al., 2014), tidyverse (Wickham et al., 2019), and patchwork packages (Pedersen, 2024).

Stata/IC 14.2 (StataCorp LLC, College Station, TX, USA) was utilized to summarize the percent recovery (i.e., bacterial isolation rate) by year, month, province, DOF when sampled, and the categories of resistance phenotypes for AMDs. Univariable analysis was performed to identify potential associations with classifying isolates as resistant using a population average logit model with feedlot as a random effect (xtlogit, StataCorp LLC, College Station, TX, USA). Separate models were used in estimating resistance prevalence for each of the antimicrobial drugs that were tested. Only univariable associations were explored; isolates with missing information for the predictor variable were excluded for that analysis only. Variables examined included the province, quarter of the year when the second sampling occurred, sampling timepoint (arrival vs. rehandling), animal age (calf vs. yearling), BRD risk category (high vs. low), and DOF category (20–40, 41–60, 61–80, 81–100, 101–120) at the time the second sample was collected. BRD risk categories were assigned by the supervising veterinarian considering age (calf or yearling), body weight (frequently representative of age), method of procurement (sale barn or ranch direct), amount of commingling before arrival, overall health assessments for the group, vaccination history, and likelihood or history of AMD exposures before arrival. High-risk cattle were typically younger, smaller, and more likely to have experienced commingling than low-risk cattle.

## 3 Results

### 3.1 Study population

**Figure 1:**
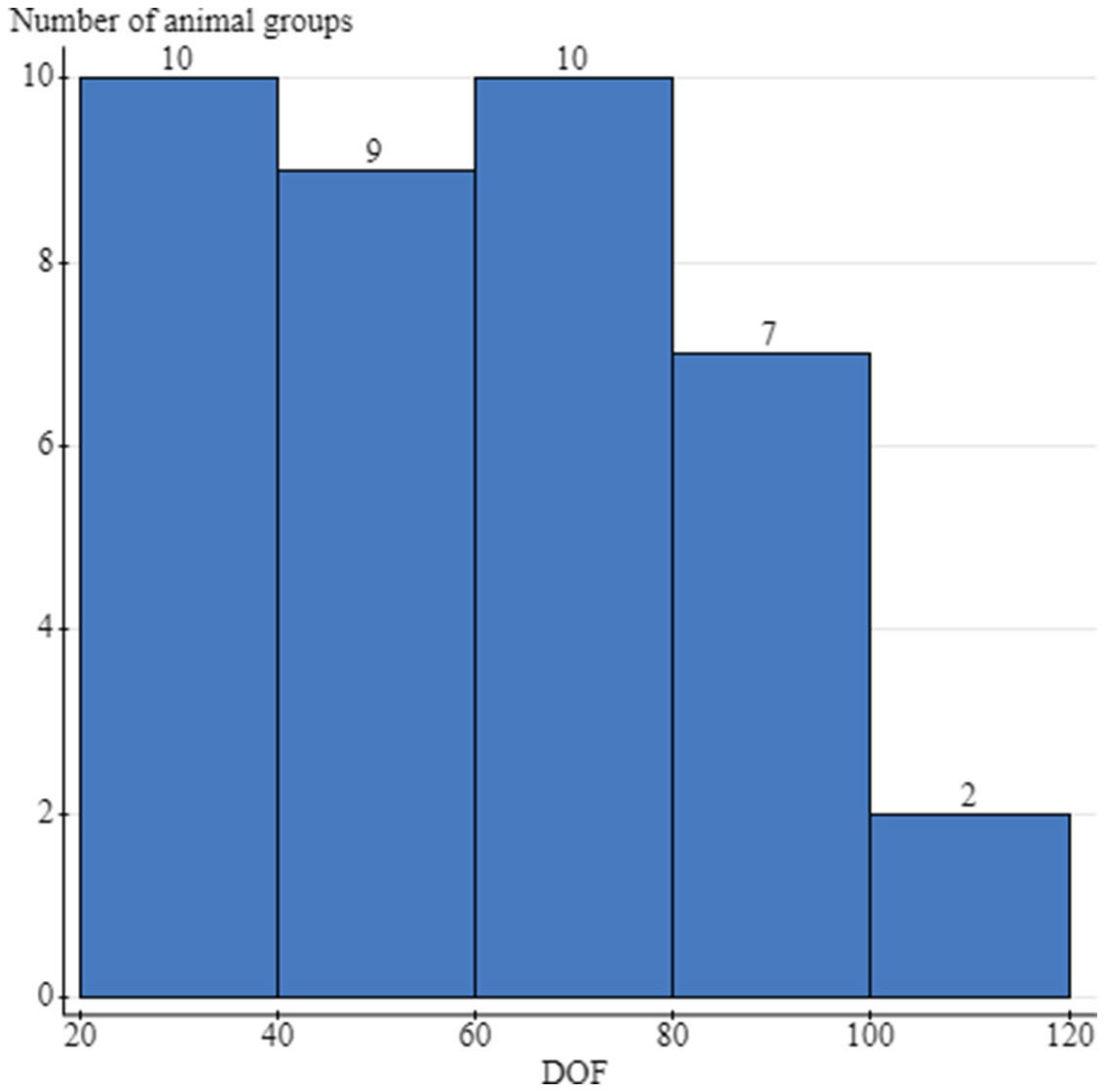
Distribution of the days on feed (DOF) at the time of the rehandling sampling and the number of cattle groups sampled per stratum. Cattle groups included the individual animals that were sampled at either arrival processing or the second rehandling timepoint, and were housed together during this timeframe. The same group of cattle was sampled at both timepoints, but not necessarily the same animals.

In 2019, 21 feedlots and 21 cattle housing groups (1 group per feedlot) were enrolled, and 608 animals were sampled. Seventeen groups had complete sampling (32 samples per group; 16 at arrival and 16 at rehandling) for a total of 544 samples; 3 groups only had the 16 arrival samples (48 total samples), and 1 group only had 16 samples collected at rehandling. In the 2020 sampling year, 26 feedlots and 26 groups (1 group/feedlot) were enrolled, and 784 cattle were sampled. Twenty-three groups had complete sampling (32 samples per group; 16 at arrival and 16 at rehandling), contributing 736 samples. One group only had the 16 arrival samples collected, and 2 groups only had rehandling samples collected, contributing 32 samples. Weight data were available for 608 animals in 2019 and 736 animals in 2020, with 48 missing values from 3 enrollment groups. Upon arrival, weights ranged from 139.2 to 503.5 kg (mean 337.7 kg, median 337 kg), and at rehandling, weights ranged from 181.4 to 635.9 kg (mean 430.6 kg, median 454.5 kg). Animal age was recorded for 98.9% (1,376/1392) of the sampled animals; 51.1% (704/1376) of these animals were classified as calves (i.e., less than 1 year of age), and 48.8% (672/1376) were classified as yearlings. Forty-seven percent (640/1360) of animals were classified as high-risk for BRD, and 53% (720/1360) were classified as low-risk. Days on feed at the time of rehandling were provided for 76.2% (16/21) of the participating feedlots in 2019 and 84.6% (22/26) in 2020, which ranged from 20 to 104 days, with a mean of 59.8 days and a median of 62 days (Figure 1).

### 3.2 Recovery rates of *Mannheimia haemolytica*, *Pasteurella multocida*, and *Histophilus somni*

A total of 608 NP swabs were collected between August 2019 and February 2020 (year 1) from 21 enrolled groups, and 784 samples were collected from 26 enrolled groups between June 2020 and February 2021 (year 2; Table 1). Overall recovery of BRD pathogens was highest for *P. multocida* (27.4%; 381/1392), followed by *H. somni* (9.0%; 125/1392) and *M. haemolytica* (8.6%; 119/1392) (Table 1). At arrival sampling, *P. multocida*, *M. haemolytica*, and *H. somni* isolates were recovered from 14.3% (199/1392), 4.1% (57/1392), and 2.2% (30/1392) of calves, respectively. At rehandling, the corresponding prevalences were 13.1% (182/1392), 4.5% (62/1392), and 6.8% (95/1392), respectively. There was variation in the recovery of BRD pathogens by province, which may have partly been attributable to the smaller number of samples and recovered isolates in cattle outside of Alberta (Table 1) and therefore results should be interpreted with caution.

**Table 1:** The number of samples collected and bacteria isolated, by study year and province.

Year	Province	Samples collected	Numbers of isolates (% recovery)		
			*M. haemolytica*	*P. multocida*	*H. somni*
2019	Alberta	496	35 (7.1)	150 (30.2)	51 (10.3)
	Saskatchewan	48	2 (4.2)	14 (29.2)	4 (8.3)
	Ontario	64	9 (14.1)	16 (25.0)	5 (7.8)
	Subtotal	608	46 (7.6)	180 (29.6)	60 (9.9)
2020	Alberta	480	46 (9.6)	134 (27.9)	47 (9.8)
	Saskatchewan	64	10 (15.6)	13 (20.3)	8 (12.5)
	Ontario	240	17 (7.1)	54 (22.5)	10 (4.2)
	Subtotal	784	73 (9.3)	201 (25.6)	65 (8.3)
	Total	1,392	119 (8.5)	381 (27.4)	125 (9.0)

Feedlot level prevalence also varied considerably by organism and year (Table 2). In 2019, 95% (20/21) of feedlots had cattle test positive for at least one of the organisms of interest. *M. haemolytica* was recovered from 71.4% (15/21) and 76.9% (20/26) of feedlots in 2019 and 2020, respectively. *Mannheimia haemolytica* was not recovered from any cattle in 2 feedlots in either year. *Pasteurella multocida* was routinely recovered from cattle in all feedlots, except in 1 feedlot in 2019, where this agent was not recovered from any cattle. In contrast, 47.6% (10/21) and 46.2% (12/26) of feedlots in 2019 and 2020, respectively, had no cattle that were culture-positive for *H. somni*. *Histophilus somni* was not recovered from any cattle in 5 of these feedlots in both years.

**Table 2:** Numbers of samples collected and bacteria isolated, by study year and feedlot.

Year	Feedlot^a^	Samples collected	Number of isolates (% recovery)		
			*M. haemolytica*	*P. multocida*	*H. somni*
2019^a^	1	32	3 (9.4)	14 (43.8)	12 (37.5)
	2	32	2 (6.3)	13 (40.6)	9 (28.1)
	3	32	3 (9.4)	8 (25.0)	12 (37.5)
	4	16	4 (25.0)	4 (25.0)	0
	5	32	1 (3.1)	15 (46.9)	8 (25.0)
	6	32	4 (12.5)	4 (12.5)	5 (15.6)
	7	32	2 (6.3)	14 (43.8)	4 (12.5)
	8	32	8 (25.0)	7 (21.9)	4 (12.5)
	23	32	1 (3.1)	4 (12.5)	0
	24	32	1 (3.1)	6 (18.8)	0
	25	32	12 (37.5)	15 (46.9)	0
	26	32	2 (6.3)	13 (40.6)	0
	27	32	0	6 (18.8)	0
	28	32	1 (3.1)	3 (9.4)	0
	29	32	0	14 (43.8)	1 (3.1)
	30	32	0	14 (43.8)	0
	31	32	0	15 (46.9)	1 (3.1)
	32	32	1 (3.1)	2 (6.3)	3 (9.4)
	33	16	0	0	0
	35	16	1 (6.3)	8 (50.0)	1 (6.3)
	36	16	0	1 (6.3)	0
2020	1	16	4 (25.0)	9 (56.3)	5 (31.3)
	3	32	2 (6.3)	5 (15.6)	0
	4	32	1 (3.1)	6 (18.8)	0
	5	32	3 (9.4)	15 (46.9)	4 (12.5)
	6	32	2 (6.3)	8 (25.0)	6 (18.8)
	7	32	6 (18.8)	11 (34.4)	8 (25.0)
	8	32	2 (6.3)	9 (28.1)	7 (21.9)
	23	32	0	14 (43.8)	2 (6.3)
	24	32	2 (6.3)	14 (43.8)	2 (6.3)
	25	32	10 (31.3)	7 (21.9)	0
	26	32	6 (18.8)	10 (31.3)	3 (9.4)
	27	32	0	2 (6.3)	1 (3.1)
	28	32	0	3 (9.4)	0
	29	32	3 (9.4)	4 (12.5)	0
	30	32	2 (6.3)	10 (31.3)	7 (21.9)
	31	32	8 (25.0)	10 (31.3)	3 (9.4)
	32	32	2 (6.3)	11 (34.4)	5 (15.6)
	33	32	4 (12.5)	2 (6.3)	0
	34	32	11 (34.4)	8 (25.0)	3 (9.4)
	35	32	2 (6.3)	6 (18.8)	0
	36	32	0	7 (21.9)	0
	37	32	0	6 (18.8)	0
	38	32	1 (3.1)	6 (18.8)	0
	40	16	1 (6.3)	1 (6.3)	0
	41	32	0	11 (34.4)	0
	42	16	1 (6.3)	6 (37.5)	9 (56.3)
Total		1,392	119 (8.6%)	381 (27.4%)	125 (9.0%)

a Samples were not collected from Feedlots 34, 37, 38, 40, 41 and 42 in 2019, and in Feedlot 2 in 2020.

Across the combined study years, the proportion of samples from which any BRD organism was recovered was highest in September, followed by November and January (Table 3).

**Table 3:** Numbers of isolates and samples by calendar month when cattle were sampled over the two-year study period.

Month	Number of isolates (% of samples)				Total samples
	*M. haemolytica*	*P. multocida*	*H. somi*	No isolates	
January	15 (8.5)	44 (25.0)	36 (20.4)	81 (46.0)	176
February	1 (10.0)	4 (40.0)	0	5 (50.0)	10
March	1 (25.0)	1 (25.0)	0	2 (50.0)	4
August	5 (18.5)	9 (33.3)	0	13 (48.0)	27
September	7 (10.0)	21 (30.0)	11 (15.7)	31 (44.3)	70
October	32 (10.7)	115 (38.3)	12 (4)	141 (47.0)	300
November	32 (9.6)	114 (34.2)	34 (10.2)	153 (45.9)	333
December	26 (10.7)	73 (29.9)	32 (13.1)	113 (46.3)	244
Total	119 (10.2)	381 (32.7)	125 (10.7)	539 (46.3)	1,164

### 3.3 Antimicrobial susceptibility testing

Among the isolates tested, 73.6% (95% CI 63.3–81.9%) of *M. haemolytica*, 67.4% (95% CI 55.2–77.6%) of *P. multocida*, and 62.1% (95% CI 47.0–75.2%) of *H. somni* isolates were susceptible to all tested AMDs with an available CLSI breakpoint. The percentage of isolates susceptible to all AMD tested decreased between arrival and rehandling for *M. haemolytica* [arrival 86.2% (95% CI 69.8–94.4%), rehandling 60.2% (95% CI 44.7–73.8%)] and *P. multocida* [arrival 80.4% (95% CI 64.4–90.3%) rehandling 49.6% (95% CI 34.0–65.3%)]. Conversely, the percentage of *H. somni* isolates susceptible to all AMD tested increased between arrival 46.7% (95% CI 12.2–84.6%) and rehandling 65.1% (95% CI 52.3–76.0%).

### 3.4 Mannheimia haemolytica

**Figure 2:**
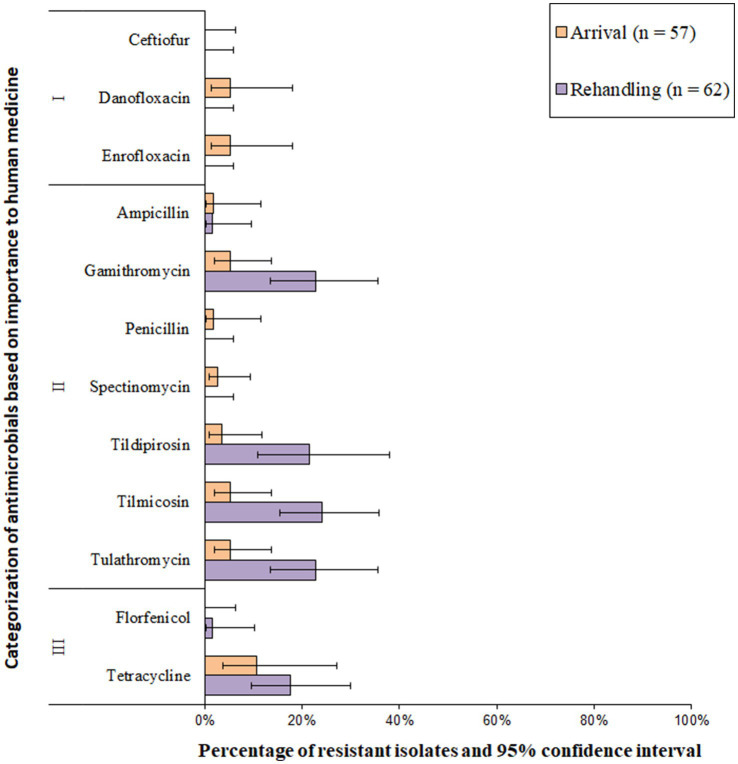
Prevalence of antimicrobial resistance in *Mannheimia haemolytica* isolates, by sampling timepoint (arrival and rehandling) in 2020 and 2021. Point estimates and 95% confidence intervals were obtained from GEE modeling, accounting for the hierarchical population structures.

Resistance to the macrolide class of antimicrobials was the most common resistance phenotype detected in *M. haemolytica* isolates (Figure 2 and Supplementary Figure S1). All isolates (*n* = 119) were susceptible to ceftiofur and florfenicol and exhibited very low resistance prevalence (0.1 to 1%) to penicillin and spectinomycin. A low prevalence of resistance (>1.0 to 10.0%) was identified for ampicillin, danofloxacin, and enrofloxacin (Figure 2). Moderate prevalence (>10.0 to 20.0%) or high prevalence (>20.0 to 50.0%) of resistance was seen for tildipirosin, gamithromycin, tulathromycin, and tilmicosin at both sampling timepoints (Figure 2). Overall, 2.4% (95% CI 0.8–7.1%) of the isolates were resistant to ≥3 AMD classes; 3.9% (95% CI 1.3–11.4%) at arrival, and 1.6% (95% CI 0.2–10.1%) at rehandling. The most common resistance pattern at rehandling was gamithromycin-tetracycline-tildipirosin-tilmicosin-tulathromycin (6.9%; 8/119).

**Figure 3:**
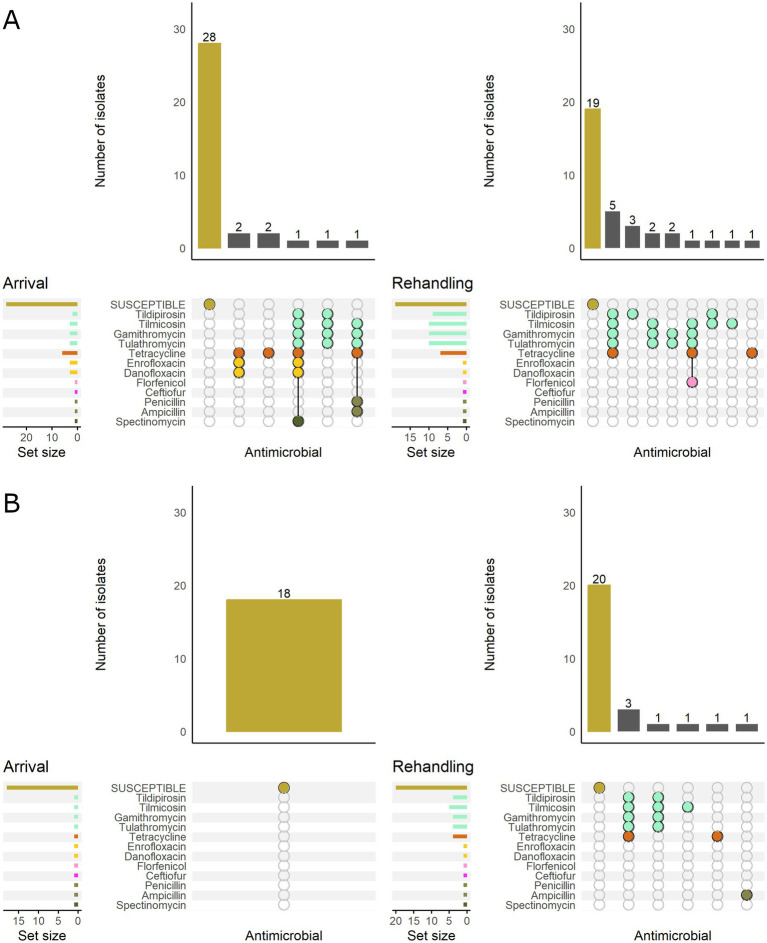
UpSet plots characterizing the intersection of antimicrobial resistance in *M. haemolytica* isolates collected from feedlot cattle and numbers of isolates with specific resistance patterns, by age and sampling timepoint. The horizontal bars at the left represent the total number of isolates within each set, and vertical bars represent the size of the intersections between the sets with resistance patterns denoted by the dots. Dots of the same color represent drugs in the same antimicrobial drug class. **(A)** Isolates from cattle <1 yr. old (*n* = 35 isolated from arrival samples and *n* = 35 isolates from rehandling). **(B)** Isolates from yearling cattle (*n* = 18 arrival, *n* = 27 at rehandling).

Based on the classification of animal age that was supplied by the supervising veterinarians, there was a marked difference in the resistance patterns between calves and yearlings (Figure 3). At arrival, for *M. haemolytica,* all isolates recovered from yearlings were pansusceptible (susceptible to all antimicrobials tested), whereas in calves, 20.0% (7/35) of isolates were resistant to ≥1 AMD class. At rehandling, however, only 25.9% (7/27) of isolates from yearlings were resistant to ≥1 AMD class compared to 45.7% (16/35) of isolates recovered from calves.

**Figure 4:**
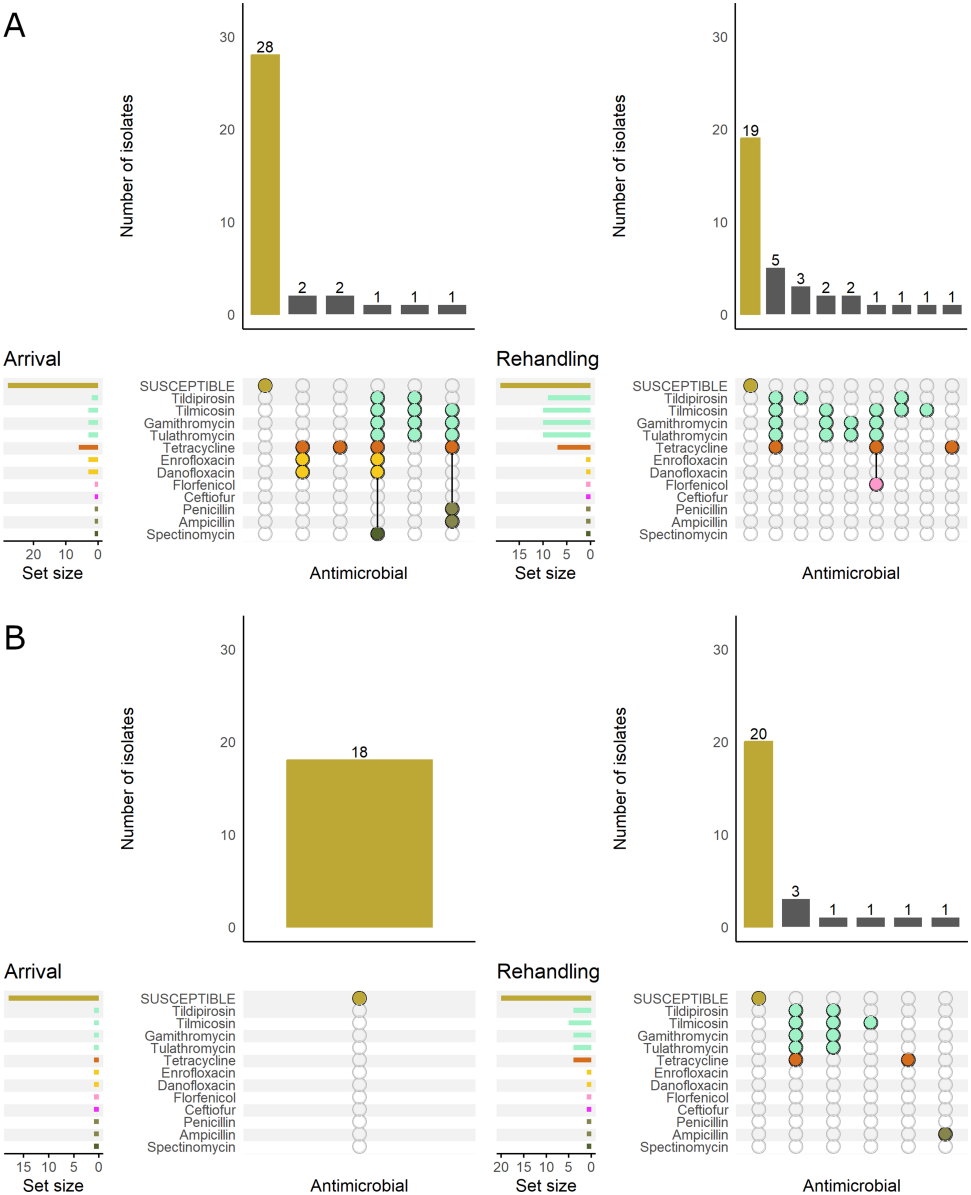
UpSet plots characterizing the intersection of antimicrobial resistance in *M. haemolytica* isolates collected from feedlot cattle and numbers of isolates with specific resistance patterns, by bovine respiratory disease (BRD) risk category and sampling timepoint. The horizontal bars at the left represent the total number of isolates within each set, and vertical bars represent the size of the intersections between the sets with resistance patterns denoted by the dots. Dots of the same color represent drugs in the same antimicrobial drug class. **(A)** Isolates from cattle with BRD risk (*n* = 33 isolated from arrival samples and *n* = 30 isolates from rehandling). **(B)** Isolates from cattle with Low BRD risk (*n* = 20 arrival, *n* = 32 at rehandling).

When *M. haemolytica* isolates were stratified by BRD risk category, isolates from low-risk cattle were all susceptible at arrival. In contrast, 21.2% (7/33) of the isolates recovered from high-risk cattle were resistant to ≥1 AMD class (Figure 4). At rehandling, 21.8% (7/32) of the isolates recovered from low-risk cattle were resistant to ≥1 AMD class, whereas 53.3% (16/30) of isolates from high-risk cattle were resistant.

### 3.5 Pasteurella multocida

**Figure 5:**
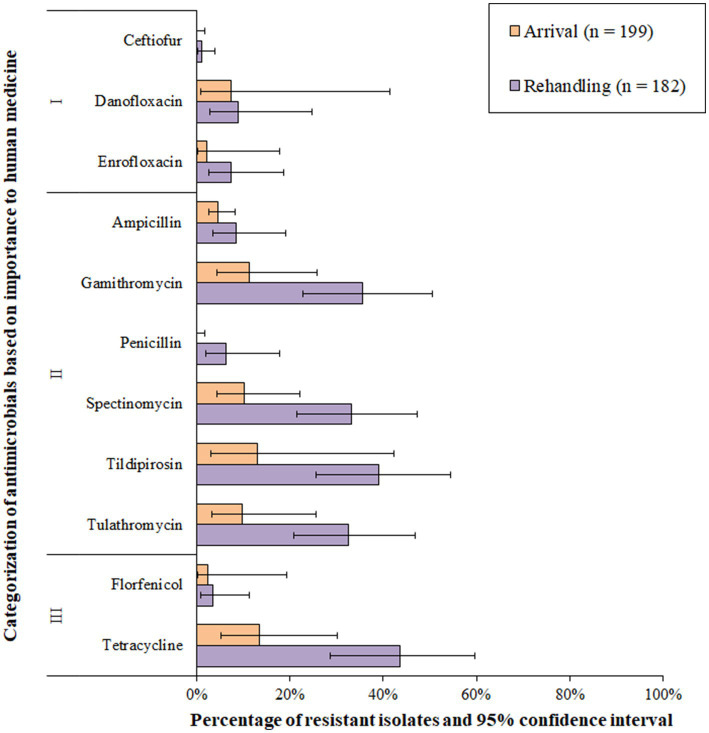
Prevalence of antimicrobial resistance in *Pasteurella multocida* isolates, by sampling timepoint (arrival and rehandling) in 2020 and 2021. Point estimates and 95% confidence intervals were obtained from GEE modeling, accounting for the hierarchical population structures.

Resistance to Category I antimicrobials was detected in less than 10% of the total isolates; resistance in this category was most often linked to the fluoroquinolone class (Figure 5 and Supplementary Table S2). Resistance prevalence for ceftiofur was very low (0.1–1.0%) in *P. multocida* isolates, whereas the resistance prevalences for enrofloxacin, penicillin, florfenicol, ampicillin, and danofloxacin were all low (>1 to 10%, Figure 5). Moderate (10–20%) or high (20–50%) resistance prevalences were identified for spectinomycin, tulathromycin, gamithromycin, tildipirosin, and tetracycline at both sampling times (Figure 5). Multidrug resistance (≥3 AMD classes) was identified in 21.3% (95% CI 12.5–33.9%) of all the isolates. Multidrug resistance increased between arrival (10.7, 95%CI 3.3–29.3%) and rehandling (36.5, 95% CI 23.8–51.5%). A single isolate recovered at rehandling was resistant to 5 AMD classes, including betalactams, fluoroquinolones, phenicols, macrolides, and aminocyclitols and (phenotypic resistance ampicillin-ceftiofur-danofloxacin-enrofloxacin-florphenicol-gamithromycin-spectinomycin-tildipirosin-tulathromycin). The most common pattern among all resistant isolates was gamithromycin-spectinomycin-tetracycline-tildipirosin-tulathromycin (13.9%, 53/381).

**Figure 6:**
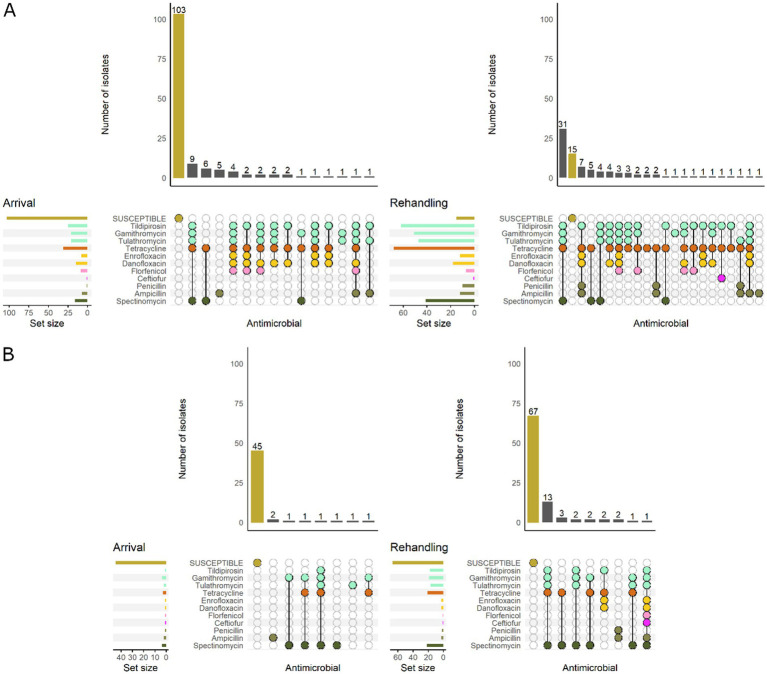
UpSet plots characterizing the intersection of antimicrobial resistance in *P. multocida* isolates collected from feedlot cattle and numbers of isolates with specific resistance patterns, by age and sampling timepoint. The horizontal bars at the left represent the total number of isolates within each set, and vertical bars represent the size of the intersections between the sets with resistance patterns denoted by the dots. Dots of the same color represent drugs in the same antimicrobial drug class. **(A)** Isolates from cattle <1 yr. old (*n* = 141 isolated from arrival samples and *n* = 89 isolates from rehandling). **(B)** Isolates from yearling cattle (*n* = 53 arrival, *n* = 93 at rehandling).

Most *P. multocida* isolates recovered from calves and yearlings were pansusceptible at arrival sampling (Figure 6). However, among resistant isolates, the phenotypic patterns were more variable in calves and generally included more AMD classes regardless of sampling timepoint. The most common pattern detected in calves included resistance to macrolides, tetracyclines, and aminocyclitols (Figure 6).

**Figure 7:**
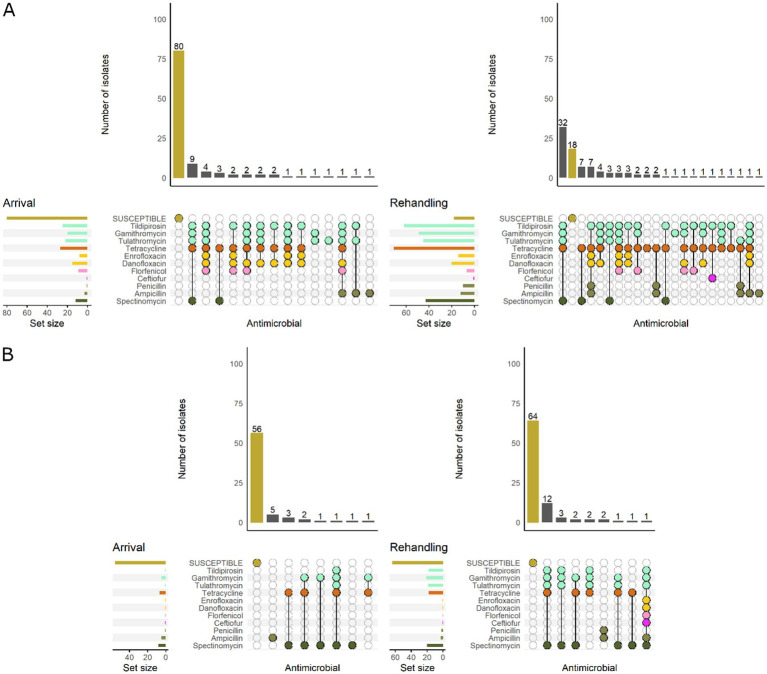
UpSet plots characterizing the intersection of antimicrobial resistance in *P. multocida* isolates collected from feedlot cattle and numbers of isolates with specific resistance patterns, by bovine respiratory disease (BRD) risk category and sampling timepoint. The horizontal bars at the left represent the total number of isolates within each set, and vertical bars represent the size of the intersections between the sets with resistance patterns denoted by the dots. Dots of the same color represent drugs in the same antimicrobial drug class. **(A)** Isolates from cattle with High BRD risk (*n* = 111 isolated from arrival samples and *n* = 94 isolates from rehandling). **(B)** Isolates from cattle with Low BRD risk (*n* = 70 arrival, *n* = 88 at rehandling).

When comparing low and high-BRD-risk cattle, the high-risk results demonstrate a similar pattern to that observed in calves, while the low-risk cattle tended to have patterns similar to those detected in yearlings (Figure 7).

### 3.6 Histophilus somni

**Figure 8:**
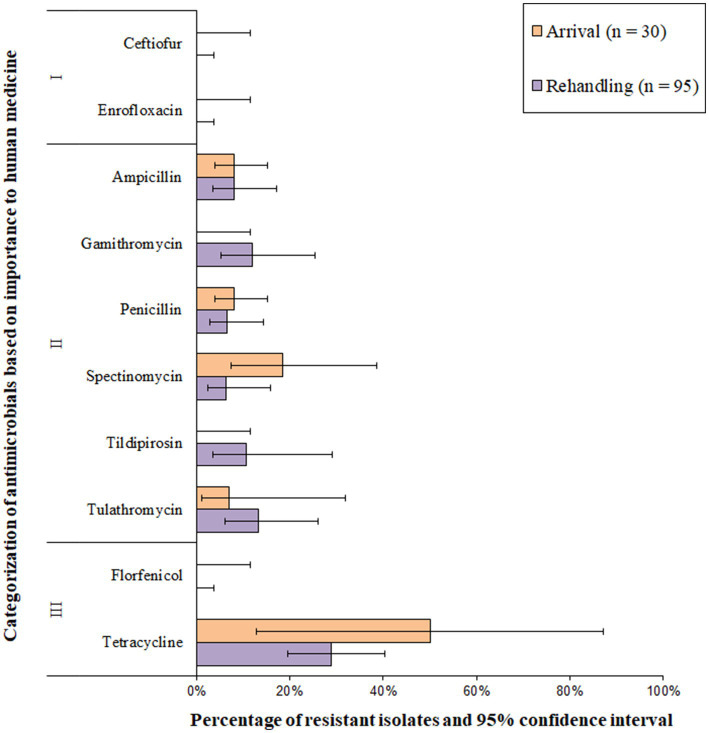
Prevalence of antimicrobial resistance in *Histophilus somni* isolates, by sampling timepoint (arrival and rehandling) in 2020 and 2021. Point estimates and 95% confidence intervals were obtained from GEE modeling, accounting for the hierarchical population structures.

*Histophilus somni* isolates were most commonly resistant to tetracyclines, followed by macrolides (Figure 8 and Supplementary Table S3). All *H. somni* isolates (*n* = 125) were susceptible to ceftiofur, enrofloxacin, and florfenicol. A low prevalence (>1.0 to 10.0%) of resistance was observed for penicillin, ampicillin, tildipirosin, gamithromycin, and spectinomycin at arrival (Figure 8). Moderate resistance prevalences (10–20%) were observed for gamithromycin, tildipirosin, and tulathromycin at rehandling, and moderate or high resistance prevalence was observed for tetracycline at both sampling times (Figure 8). Two of the 125 *H. somni* isolates (1.6%) were resistant to 4 AMD classes, including betalactams, macrolides, aminocyclitols, and tetracycline. Resistance to ≥3 AMD classes was identified in 3.4% (95% CI 0.7–14.8%) of the isolates. Overall, 3.3% (95% CI 0.5–19.6%) of arrival isolates and 4.5% (95% CI 1.1–16.7%) of rehandling isolates were resistant to ≥3 AMD classes.

**Figure 9:**
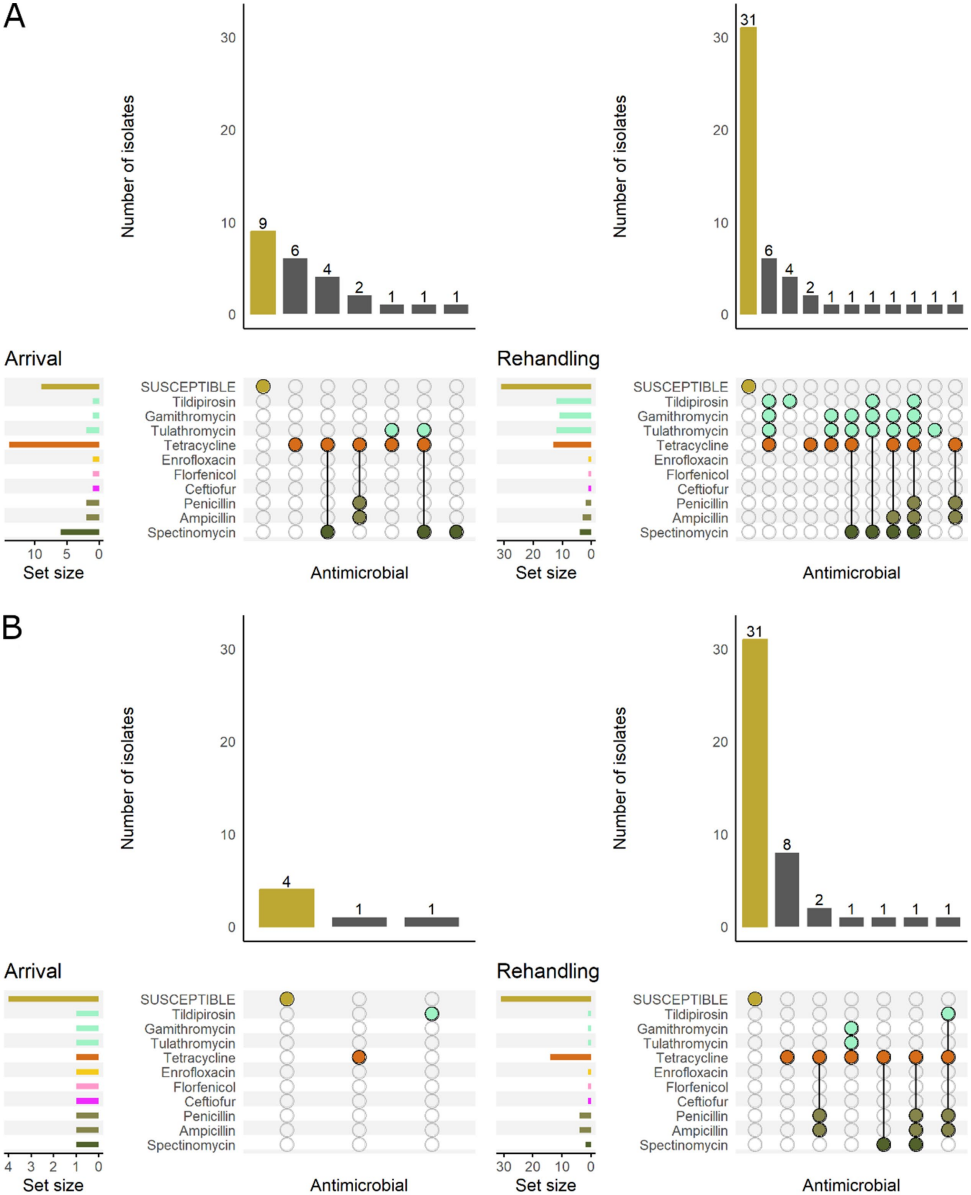
UpSet plots characterizing the intersection of antimicrobial resistance in *H. somni* isolates collected from feedlot cattle and numbers of isolates with specific resistance patterns, by age and sampling timepoint. The horizontal bars at the left represent the total number of isolates within each set, and vertical bars represent the size of the intersections between the sets with resistance patterns denoted by the dots. Dots of the same color represent drugs in the same antimicrobial drug class. **(A)** Isolates from cattle <1 yr old (*n* = 24 isolated from arrival samples and n = 50 isolates from rehandling). **(B)** Isolates from yearling cattle (*n* = 6 arrival, *n* = 45 at rehandling).

Isolates recovered from yearling cattle at arrival were most commonly pansusceptible (Figure 9). The two resistant arrival *H. somni* isolates collected from yearlings were each resistant to a single AMD class. Of the isolates recovered from calves at arrival, only 37.5% (9/24) were pansusceptible. At rehandling, resistant isolates recovered from either yearling cattle or calves tended to be resistant to several AMD classes. However, in general, isolates obtained from yearling cattle at rehandling were resistant to fewer AMD’s than isolates obtained from calves at the same timepoint (Figure 9).

**Figure 10:**
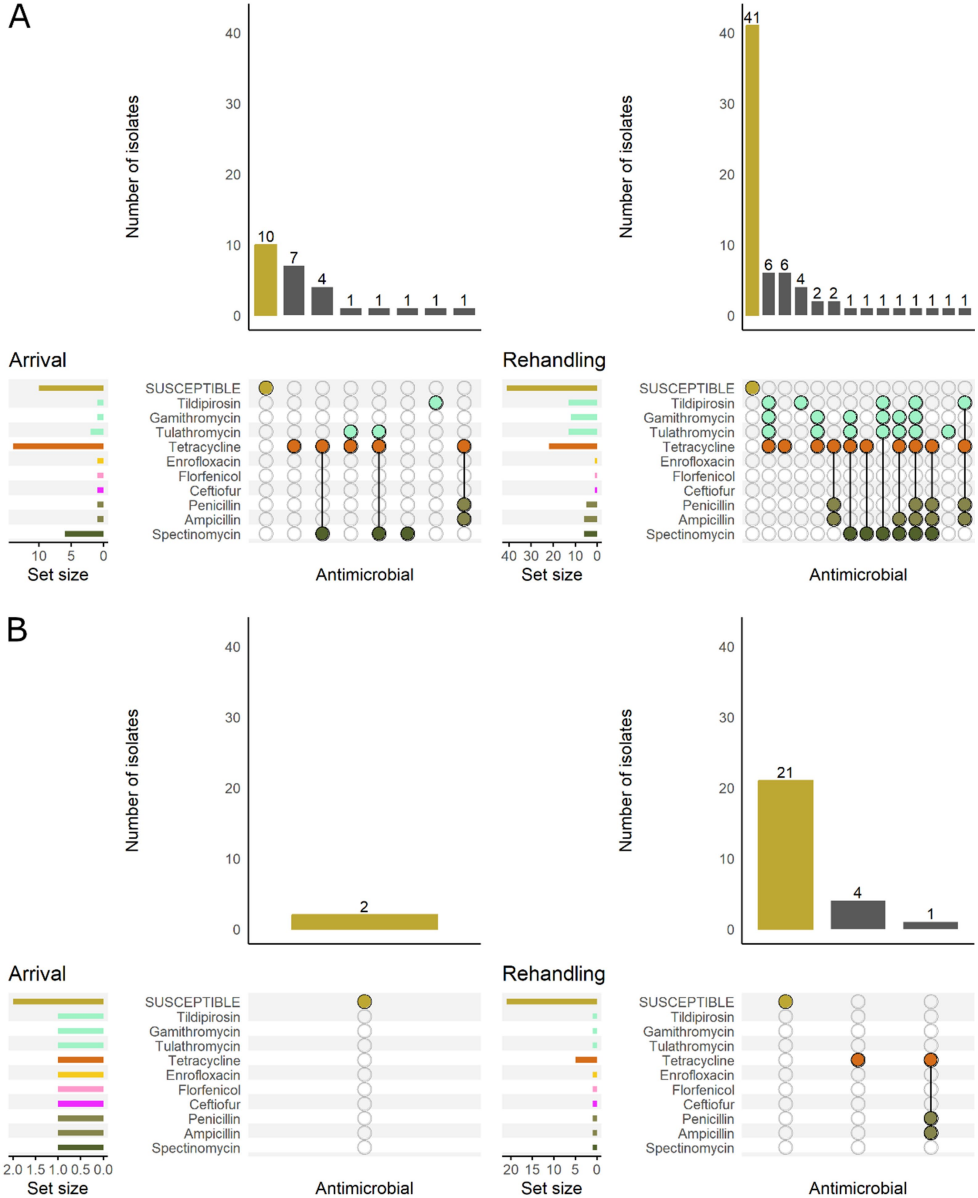
UpSet plots characterizing the intersection of antimicrobial resistance in *H. somni* isolates collected from feedlot cattle and numbers of isolates with specific resistance patterns, by bovine respiratory disease (BRD) risk category and sampling timepoint. The horizontal bars at the left represent the total number of isolates within each set, and vertical bars represent the size of the intersections between the sets with resistance patterns denoted by the dots. Dots of the same color represent drugs in the same antimicrobial drug class. **(A)** Isolates from cattle with High BRD risk (*n* = 26 isolated from arrival samples and *n* = 69 isolates from rehandling). **(B)** Isolates from cattle with Low BRD risk (*n* = 2 arrival, *n* = 26 at rehandling).

All *H. somni* isolates recovered from low-risk cattle were pansusceptible at arrival (Figure 10), and most (80%, 21/26) isolates were also pansusceptible when recovered at rehandling. In high-risk cattle, only 38% (10/26) of the arrival isolates were pansusceptible, while 59% (41/69) were pansusceptible at rehandling. Resistant isolates from low-risk cattle at rehandling only demonstrated resistance to 1–2 AMD classes. In contrast, isolates recovered from high-risk cattle more commonly had resistance to ≥2 AMD classes and had greater diversity in resistance patterns regardless of sampling time (Figure 10).

### 3.7 Risk factor analysis

Risk factors associated with the identification of resistance varied between BRD pathogens. Only 3 of the 6 risk factors explored were statistically significantly associated with AMR, including sampling timepoint (arrival vs. rehandling), animal age category (calf vs. yearling), and DOF category (20–40, 41–60, 61–80, 81–100, 101–120). In general, isolates recovered at arrival were less likely to be resistant than those recovered later in the feeding period. For the *M. haemolytica* and *P. multocida* isolates, there was significantly less resistance to all of the tested macrolides with CLSI breakpoints at arrival than at rehandling (Table 4). Among isolates recovered at arrival, for *P. multocida* only, resistance to ampicillin, danofloxacin, enrofloxacin, tetracycline, and spectinomycin was less likely compared to isolates obtained at rehandling (Table 4). No significant differences were noted between the two timepoints for *H. somni*.

**Table 4:** Odds ratios and 95% confidence intervals comparing the prevalence of antimicrobial resistance in *M. haemolytica*, *P. multocida* and *H. somni* between arrival and rehandling sampling time points.

Resistance outcome variable	*M. haemolytica*			*P. multocida*			*H. somni*		
	Odds ratio	95% CI^*^	*p* value	Odds ratio	95% CI	*p* value	Odds ratio	95% CI	*p* value
Ampicillin	0.92	0.06–15.02	0.95	2.74	1.03–7.26	0.04	1.63	0.15–17.95	0.69
Penicillin	^a^	–	–	^a^	–	–	0.97	0.11–8.79	0.98
Ceftiofur	^a^	–	–	^a^	–	–	^a^	–	–
Danofloxacin	^a^	–	–	5.44	1.50–19.70	0.01	3.17	0.23–43.17	0.49
Enrofloxacin	^a^	–	–	3.81	1.30–11.13	0.01	^a^	–	–
Flofenicol	^a^	–	–	0.93	0.28–3.05	0.90	^a^	–	–
Gamithromycin	6.13	1.33–28.22	0.02	8.99	4.37–18.48	<0.0001	^a^	–	–
Spectinomycin	^a^–	^a^–	^a^–	7.29	3.63–14.61	<0.0001	0.40	0.08–2.05	0.27
Tetracycline	2.12	0.57–7.73	0.26	16.37	7.68–34.88	<0.0001	1.10	0.28–4.26	0.89
Tildipirosin	15.86	1.71–147.0	0.02	23.41	9.62–56.97	<0.0001	2.87	0.24–33.86	0.40
Tilmicosin	5.75	1.57–21.10	0.01	^b^	–	–	^b^	–	–
Tulathromycin	6.13	1.33–28.22	0.02	11.20	5.41–23.18	<0.0001	2.49	0.37–16.85	0.35

95%CI = 95% confidence interval.

a The model failed to converge due to the low prevalence of antimicrobial resistance.

b There are no CLSI approved breakpoints for *P. multocida* and *H. somni* for Tilmicosin.

Significant differences in resistance were detected for *P. multocida* and *H. somni* isolates obtained from calves vs. yearlings (Table 5). The odds of resistance to tulathromycin were about 12 times greater in *H. somni* isolates obtained from yearlings (OR = 12.0, 95% CI = 1.03–140.4, *p* = 0.05; Table 5). Similarly, *P. multocida* isolates from calves were more likely to be resistant to gamithromycin, spectinomycin, tetracycline, tildipirosin, and tulathromycin than isolates obtained from yearlings (Table 5). *P. multocida*, isolates obtained from samples collected between 81 and 100 DOF were less likely to be resistant to spectinomycin than isolates from samples collected between 20 and 40 DOF (Table 6). Isolates from samples obtained between 61 and 80 DOF or between 81 and 100 DOF were less likely to be resistant to tetracycline, tildipirosin, and tulathromycin than isolates from samples collected between 20 and 40 DOF.

**Table 5:** Odds ratios and 95% confidence intervals comparing the prevalence of antimicrobial resistance in *M. haemolytica*, *P. multocida*, and *H. somni* between calves and yearlings.

Resistance outcome variable	*M. haemolytica*			*P. multocida*			*H. somni*		
	Odds ratio	95% CI^*^	*p* value	Odds ratio	95% CI	*p* value	Relative risk	95% CI	*p* value
Ampicillin	0.64	0.03–10.50	0.75	2.17	0.70–6.81	0.18	0.81	0.17–3.78	0.79
Penicillin	^a^	–	–	0.79	0.06–11.17	0.86	0.66	0.15–2.98	0.59
Ceftiofur	^a^	–	–	0.63	0.04–10.20	0.75	^a^	–	–
Danofloxacin	^a^	–	–	1.62	0.09–28.39	0.74	0.69	0.04–11.21	0.79
Enrofloxacin	^a^	–	–	1.70	0.13–21.52	0.68	^a^	–	–
Flofenicol	^a^	–	–	2.48	0.12–50.75	0.56	^a^	–	–
Gamithromycin	5.00	0.63–39.73	0.13	3.58	1.19–10.81	0.02	8.85	0.73–107.10	0.09
Spectinomycin	^a^	–	–	2.81	1.02–7.76	0.05	3.64	0.46–28.76	0.22
Tetracycline	4.33	0.64–29.19	0.13	8.19	2.52–26.61	<0.0001	1.34	0.25–7.21	0.73
Tildipirosin	6.60	0.39–112.76	0.19	5.90	1.85–18.74	0.003	6.91	0.47–102.18	0.16
Tilmicosin	2.01	0.54–7.51	0.30	^b^	–	–	^b^	–	–
Tulathromycin	5.00	0.63–39.73	0.13	3.98	1.33–11.85	0.01	12.03	1.03–140.42	0.05

Yearling was the reference group for the analysis. CI = 95% confidence interval.

a The model failed to converge due to the low level of antimicrobial resistance.

b There are no CLSI approved MIC breakpoints for *P. multocida* and *H. somi* for this Tilmicosin.

**Table 6:** Odds ratios and 95% confidence intervals comparing the prevalence of antimicrobial resistance in *Pateurella multocida* obtained at rehandling, by the days on feed at the time of sampling.

Antimicrobial drug	Days on feed	Number of isolates	Odds ratio	95% CI	*p* value
Spectinomycin	20–40		Reference		
	41–60	35	1.01	0.07–14.27	1.00
	61–80	59	0.16	0.02–1.41	0.10
	81–100	31	0.03	0.003–0.40	0.01
	101–120	9	3.40	0.04–257.11	0.58
Tetracycline	20–40		Reference		
	41–60	35	1.58	0.15–16.21	0.70
	61–80	59	0.05	0.003–0.62	0.02
	81–100	31	0.13	0.02–0.97	0.05
	101–120	9	0.11	0.001–9.00	0.33
Tilidpirosin	20–40		Reference		
	41–60	35	0.88	0.07–10.89	0.92
	61–80	59	0.02	0.001–0.51	0.02
	81–100	31	0.01	0.0001–0.23	0.01
	101–120	9	1.10	0.01–140.20	0.97
Tulathromycin	20–40		Reference		
	41–60	35	0.09	0.01–1.45	0.09
	61–80	59	0.01	0.001–0.25	0.003
	81–100	31	0.01	0.0001–0.19	0.004
	101–120	9	0.86	0.01–82.97	0.95

95%CI, 95% confidence interval.

## 4 Discussion

This study provides important information about the ability to recover three key BRD pathogens, *M. haemolytica*, *P. multocida*, and *H. somni*, and the and antimicrobial susceptibility of those isolates. Most studies of AMR in cattle have examined fecal organisms such as *E. coli*, which are not pathogens that drive AMU decisions by producers and veterinarians. It is additionally valuable that NP samples were collected from a study population that is broadly representative of feedlot cattle produced in Canada, which is a goal of the Public Health Agency of Canada’s systematic surveillance program for AMU and AMR in food animal populations. While BRD has been extensively studied, few publications have compared the recovery and AMR profiles of these populations from nasopharyngeal swabs collected from healthy feedlot cattle between arrival processing and rehandling (i.e., 20 to 120 DOF).

AMR patterns differed among the three bacterial species examined here. Over half of the BRD pathogen isolates recovered in this study were pansusceptible to all AMDs tested, but the proportion of pansusceptible isolates at feedlot arrival was lower than in comparable Canadian studies of BRD isolates (Noyes et al., 2015; Erickson et al., 2017). Conversely, there was a higher proportion of pansusceptible isolates at rehandling in this study than in Erickson et al. (2017). Very low resistance prevalence was observed for ceftiofur, a Health Canada Category I antimicrobial, in *P. multocida* isolates, and no ceftiofur resistance was identified in *M. haemolytica* and *H. somni* isolates. This is consistent with previous studies in which fewer than 1% of *Pasterellaceae* isolates reported in studies conducted in the United States and Canada exhibited ceftiofur resistance (Klima et al., 2014; Anholt et al., 2017; Timsit et al., 2017; Woolums et al., 2018; Kadlec et al., 2019). Based on these findings, ceftiofur potentially remains an efficacious option for treating BRD. However, there are vocal concerns regarding using 3^rd^ generation cephalosporins in animals due to their classification as essential for last-resort treatment for multidrug-resistant infections in humans (World Health Organization, 2019). Producers and veterinarians are frequently criticized when they use licensed antimicrobial products according to approved label requirements that contradict recommendations from prominent public health groups. This ongoing debate highlights the importance of surveillance efforts similar to those reported in this study, which provide grounding for evidence-based decisions regarding AMDs. Brault et al. (2019) reported that less than 1% of the medically important antimicrobials used in feedlot cattle are Category I antimicrobials.

Consistent with previous studies conducted in Canadian and American feedlots, *P. multocida* was recovered more frequently than *M. haemolytica* and *H. somni* (Portis et al., 2012; Guo et al., 2020; Holschbach et al., 2020; Schonecker et al., 2020; Andres-Lasheras et al., 2021). However, *M. haemolytica* and *H. somni* were recovered less frequently than previously reported (Noyes et al., 2015; Andres-Lasheras et al., 2021; Erickson et al., 2017; Wennekamp, 2020). Previous studies recovered *M. haemolytica* from 14 to 40% of cattle in large populations of feedlot cattle in Alberta (Andres-Lasheras et al., 2021) and at even higher prevalences among auction-sourced calves entering Saskatchewan feedlots (28–40%) (Erickson et al., 2017; Wennekamp, 2020). Follow-up studies are being conducted to further investigate the unexpectedly lower recovery (8.9%) of *M. haemolytica* identified in this study.

The results of this study broadly agree with the findings of comparable studies published in recent years. Resistance of BRD pathogens belonging to the family Paseurellaecae, which includes all 3 organisms targeted in this study, has most commonly been reported for macrolide and tetracycline AMD’s. In a cross-sectional survey of beef cattle, Andres-Lasheras et al. (2021) described the prevalence of resistance in *M. haemolytica* upon arrival to feedlots as being highest for oxytetracycline (10%), followed by tilmicosin (6.4%) and then ampicillin (4.6%).

A greater proportion of *P. multocida* isolates possessed an MDR phenotype at rehandling than at arrival. The most common resistance pattern in *P. multocida* isolates included macrolides, aminocyclitols, and tetracyclines. Integrative and conjugative elements (ICE) carrying multiple AMR genes have been increasingly detected in members of the *Pasteurellaceae* family (Michael et al., 2012; Eidam et al., 2015; Klima et al., 2016; Cameron et al., 2018; Stanford et al., 2020; Kadlec et al., 2019). Therefore, the MDR patterns observed might indicate the presence of ICE in these isolates. There are plans to examine a subset of isolates to explore the presence of AMR genes and mobile genetic elements. Since the literature indicates that ICE can be carried by and transferred between these *Pasteurellaceae* species (Beker et al., 2018; Klima et al., 2019; Bhatt et al., 2018), exploration of a subset of the MDR *M. haemolytica* and *H. somni* isolates will also be completed to explore possible similarities in genotypes between BRD organisms.

The ability to detect significant relationships with the factors evaluated in this study may have been limited by the relatively small number of isolates studied and the low resistance prevalence for many drugs. Despite this, animal age category, sampling time, and DOF were significant risk factors for resistance for some antimicrobials and BRD organisms. *Pasteurella multocida* isolates obtained from calves were more likely than yearlings to be resistant to the macrolide, aminocyclitol, and tetracycline classes, while *H. somni* isolates from calves were more likely to be resistant to tulathromycin than those from yearlings. Despite no significant results being detected for resistance in *M. haemolytica* isolates and animal age, the upset plots for all 3 organisms illustrated that the resistance patterns detected between calves and yearlings varied, with more diverse patterns seen in calves. Age-related differences in resistance among BRD pathogens might be related to management practices in the calves’ herds of origin or the approach to managing calves within the feedlot, but additional research is needed to explore these relationships further.

The time of sample collection (arrival vs. rehandling) was significantly related to macrolide resistance in *M. haemolytica*, and macrolide, tetracycline, and spectinomycin resistance in *P. multocida* isolates. Some of the literature suggests that developing resistance in BRD pathogens is a likely consequence of metaphylactic and therapeutic AMU (Erickson et al., 2017; Guo et al., 2020; Timsit et al., 2017). A recent report linked parenteral administration of metaphylactic macrolides to elevated MICs in BRD pathogens isolated from suckling beef calves (Nobrega et al., 2021) and feedlot cattle (Abi Younes et al., 2022). Likewise, Snyder et al. (2017) detected a rise in phenotypic tulathromycin resistance (from 3.7 to 99.2%) in *M. haemolytica* isolates from calves treated with tulathromycin in the previous 2 weeks. Administration of AMDs and timing of sample collection seems to be a factor in the AMR patterns seen based on the above literature. Unfortunately, the current study does not have AMU data matched to the NP sample collection. Therefore, the impact of AMU on the resistance patterns detected cannot be fully assessed.

The upset plots demonstrate that the BRD risk category, while not found to be a statistically significant risk factor, reflects calf and yearling AMR patterns, with high-risk cattle results resembling the calves and low-risk cattle resembling the yearlings. These findings are consistent with some of the factors that are used to categorize BRD risk in cattle. A high BRD risk is generally attributed to younger, lighter cattle, consistent with these cattle being classified as calves. Organism and sampling time also impacted the differences between calves and yearlings and BRD risk categorization.

Days on feed also impacted AMR in *P. multocida*. A significant decrease in resistance to spectinomycin, tetracycline, tildipirosin, and tulathromycin in *P. multocida* isolates in the 61–80 and the 81–100 DOF compared to 20–40 DOF. As measured in this study, the diversity in prevalence, patterns, and significant risk factors between the BRD organisms and the antimicrobial indicate that many variables can impact AMR. These findings are important to consider in reporting and future work since the population being sampled, the time of the sampling, and the organism could impact the outcomes of interest.

## 5 Conclusion

This study examined the recovery of BRD pathogens from a population of healthy feedlot cattle that was representative of most beef feedlot cattle produced in Canada. There was a general increase in resistance among isolates between arrival and rehandling, with a notable trend for increasing MDR prevalence among *P. multocida* isolates. Sampling time and animal age category were associated with an increased likelihood of isolates being resistant to certain antimicrobial drugs. The increase in resistance to macrolides and tetracyclines is consistent with recent trends in the literature. The unknown history of AMD exposures prior to feedlot arrival and during the study period is a limitation of this study, and further investigations of the impacts of the direct impacts of AMD exposures are needed. This research generates additional questions about the potential presence of ARGs and mobile genetic elements such as plasmids or ICE in these isolates. Ongoing monitoring is needed to develop strategies that promote antimicrobial stewardship, minimize AMR risk, and help ensure the continued effectiveness of antimicrobials.

## Data Availability

The datasets presented in this study can be found in online repositories. The names of the repository/repositories and accession number(s) can be found at: https://dataverse.tdl.org/dataverse/AMRprevalenceinBRDpathogens2024.
