# Supplementary material for: The prevalence and antimicrobial resistance of respiratory pathogens isolated from feedlot cattle in Canada
Source: Front Microbiol. 2025 Jan 28;16:1497402. doi: 10.3389/fmicb.2025.1497402 (PMC11810952; doi:10.3389/fmicb.2025.1497402)
Supplement: Supplementary file 1 [file Data_Sheet_1.zip › Rattanapanadda_Supplemental_Table3.22DEC2024.pdf]

Rattanapadda et al., 2025. [doi: 10.3389/fmicb.2025.1497402]

Supplemental Table 3: Distribution of minimum inhibitory concentrations (MICs) among *Histophilus somni* isolates (arrival n= 30, rehandling n= 95).

Class	Category	Antimicrobial agents	Time Point	MIC_{50}	MIC_{90}	%R	MIC Distribution (µg/mL)												
							0.125	0.25	0.5	1	2	4	8	16	32	64	128	256	512
Fluoroquinolones	I	Danofloxacin	Arv	0.12	0.185	-	27	2	1										
			Re	0.12	0.12	-	92	2	1										
	I	Enrofloxacin	Arv	0.12	0.12	0	28	1	0	1									
			Re	0.12	0.12	0	93	0	1	1									
ß-lactam	I	Ceftiofur	Arv	0.25	0.25	0		30	0										
			Re	0.25	0.25	0		94	1										
	II	Ampicillin	Arv	0.25	0.25	6.7		28	0	1	1								
			Re	0.25	0.25	7.4		88	4	2	1								
	II	Penicillin	Arv	0.12	0.185	6.7	27	1	0	0	1	1							
			Re	0.12	0.12	7	87	1	1	3	2	1							
Lincosamide	II	Clindamycin	Arv	1	2	-		0	3	22	4		1	0	0				
			Re	1	2	-		1	13	50	27		1	1	2				
Macrolides	II	Gamithromycin	Arv	1	1.5	0				27	2		1	0					
			Re	1	16	12.6				77	6		0	12					
	II	Tildipirosin	Arv	4	8	0.0				0	4	20	5	1	0				
			Re	4	32	10.5				1	25	24	32	3	10				
	II	Tilmicosin	Arv	8	16	-					0	7	16	5	2				
			Re	8	32	-					2	25	37	18	13				
	II	Tulathromycin	Arv	16	32	6.7							10	14	4	2	0		
			Re	16	128	13.7							35	27	20	1	12		
	II	Tylosin	Arv	8	16	-				0	1	11	9	8	1	0			
			Re	8	16	-				1	7	35	40	10	0	2			
Aminoglycoside	II	Gentamicin	Arv	16	32	-						0	13	4	13				
			Re	16	32	-						4	19	45	27				
	II	Neomycin	Arv	64	64	-							0		10	20			
			Re	64	64	-							1		25	69			
	III	Spectinomycin	Arv	32	128	20							0	10	13	1	6		
			Re	32	64	6.3							3	19	57	10	6		
Phenicols	III	Florfenicol	Arv	0.25	1	0		20	6	4									
			Re	0.25	0.25	0		87	6	2									
Pleuromutilin	III	Tiamulin	Arv	2	4	-			1	5	19	3	1	1	0				
			Re	2	4	-			5	36	35	15	0	0	4				
Tetracyclines	III	Tetracycline	Arv	6	16	50.0			14		0	1	3	12					
			Re	0.5	16	28.4			67		1	0	5	22					
Sulfonamides	III	Sulphadimethoxime	Arv	256	512	-												16	14
			Re	512	512	-												37	58
	III	Trimethoprim/ sulphamethoxazole	Arv	2	4	-					23	7							
			Re	2	2	-					89	6							

Abbreviations: Arv: arrival; Re: rehandling. The shaded areas indicate concentrations on the panel not tested. White cells indicate the antimicrobial concentration range tested. Values above the tested range indicate an MIC value higher than the highest concentration tested. Values corresponding to the lowest concentration tested indicated MIC values lower or equal to the lowest concentration within the range. The categorizations used were based on importance to human medicine (Health Canada, 2009). The double green and red vertical lines refer to susceptible and resistant breakpoints respectively (CLSI, 2018). MIC_{50} = antimicrobial drug concentration that inhibit 50% of the bacterial population. MIC_{90} = antimicrobial drug concentration that inhibit 90% of the bacterial population.
