# Supplementary material for: The prevalence and antimicrobial resistance of respiratory pathogens isolated from feedlot cattle in Canada
Source: Front Microbiol. 2025 Jan 28;16:1497402. doi: 10.3389/fmicb.2025.1497402 (PMC11810952; doi:10.3389/fmicb.2025.1497402)
Supplement: Supplementary file 1 [file Data_Sheet_1.zip › Rattanapanadda_Supplemental_Table2.22DEC2024.pdf]

Rattanapadda et al., 2025. [doi: 10.3389/fmicb.2025.1497402]

Supplemental Table 2: Distribution minimum inhibitory concentrations (MICs) among *Pasteurella multocida* isolates (arrival n= 199, rehandling n= 182).

Class	Category	Antimicrobial agents	Time Point	MIC_{50}	MIC_{90}	%R	MIC Distribution (µg/mL)												
							0.125	0.25	0.5	1	2	4	8	16	32	64	128	256	512
Fluoroquinolones	I	Danofloxacin	Arv	0.12	0.12	8	183	1	0	0	15								
			Re	0.12	2	12	157	3	1	1	20								
	I	Enrofloxacin	Arv	0.12	0.12	1.0	182	0	9	6	2								
			Re	0.12	1	2.7	160	1	6	10	5								
ß-lactam	I	Ceftiofur	Arv	0.25	0.25	0		191	7		0	1		0					
			Re	0.25	0.25	1		175	3		1	1		2					
	II	Ampicillin[a]	Arv	0.25	0.25	4.5		190	9	0									
			Re	0.25	0.25	8.2		167	3	12									
	II	Penicillin	Arv	0.12	0.25	0.0	146	52	1	0				0					
			Re	0.12	0.25	7	128	41	1	1				11					
Lincosamide	II	Clindamycin	Arv	32	32	-		3	0				2	20	174				
			Re	32	32	-		0	2				0	5	175				
Macrolides	II	Gamithromycin	Arv	1	16	13				158	7	2	7	25					
			Re	2	16	38.5				83	13	2	14	70					
	II	Tildipirosin	Arv	1	32	13.1				132	32	8	1		26				
			Re	2	32	44.0				63	30	5	4		80				
	II	Tilmicosin	Arv	4	32	-					27	87	44	9	32				
			Re	16	32	-					3	40	44	14	81				
	II	Tulathromycin	Arv	8	128	6.7							169	4	3	2	21		
			Re	8	128	13.7							110	2	6	3	61		
	II	Tylosin	Arv	32	64	-			1	1		1	6	20	97	73			
			Re	64	64	-			0	0		0	2	7	43	130			
Aminoglycoside	II	Gentamicin	Arv	4	8	-				3	42	126	24	1	3				
			Re	4	8	-				2	28	109	38	1	4				
	II	Neomycin	Arv	16	64	-						13	73	69	6	38			
			Re	16	64	-						3	54	37	6	82			
	III	Spectinomycin	Arv	16	128	10							5	96	76	2	20		
			Re	32	128	35							2	45	72	0	63		
Phenicols	III	Florfenicol	Arv	0.5	1	4.5		68	111	8	2	1	8	1					
			Re	0.5	1	4.4		11	125	34	1	3	4	4					
Pleuromutilin	III	Tiamulin	Arv	32	32	-			1	1	3	4	11	65	95	19			
			Re	32	64	-			1	0	0	1	3	30	106	41			
Tetracyclines	III	Tetracycline	Arv	0.5	8	17.1			150	11	2	2	18	16					
			Re	4	16	48.9			82	3	0	8	39	50					
Sulfonamides	III	Sulphadimethoxime	Arv	512	512	-												88	111
			Re	512	512	-												60	122
	III	Trimethoprim/ sulphamethoxazole	Arv	2	2	-					191	8							
			Re	2	4	-					162	20							

Abbreviations: Arv: arrival; Re: rehandling. The shaded areas indicate concentrations on the panel not tested. White cells indicate the antimicrobial concentration range tested. Values above the tested range indicate an MIC value higher than the highest concentration tested. Values corresponding to the lowest concentration tested indicated MIC values lower or equal to the lowest concentration within the range. The categorizations used were based on importance to human medicine (Health Canada, 2009). The double green and red vertical lines refer to susceptible and resistant breakpoints respectively (CLSI, 2018). MIC_{50} = antimicrobial drug concentration that inhibit 50% of the bacterial population. MIC_{90} = antimicrobial drug concentration that inhibit 90% of the bacterial population.
