# Supplementary material for: The prevalence and antimicrobial resistance of respiratory pathogens isolated from feedlot cattle in Canada
Source: Front Microbiol. 2025 Jan 28;16:1497402. doi: 10.3389/fmicb.2025.1497402 (PMC11810952; doi:10.3389/fmicb.2025.1497402)
Supplement: Supplementary file 1 [file Data_Sheet_1.zip › Rattanapanadda _Supplemental_Methods.22DEC2024.pdf]

Rattanapadda et al., 2025. [doi: 10.3389/fmicb.2025.1497402]

Supplemental Methods – Methods used in evaluating sample size and identifying the sampling frame for feedlots included in the study.

The sampling goal was to be representative of the fed cattle population in Canada. Ninety percent of Canadian fed cattle are in Alberta (64%), Ontario (19%) and Saskatchewan (7%)[Statistics Canada. *Number of cattle, by class and farm type (x 1,000)*. Statistics Canada, 2024]. Therefore these three provinces were included in the sampling frame. As detailed below, the total fed cattle to be sampled were distributed proportionally among the provinces, among the varying feedlot capacities, and among the participating clinics.

Calculation of representative sample size of cattle in feedlots (>1000 head) to estimate antimicrobial use (treatment incidence) in feedlots in Alberta, Saskatchewan, and Ontario with stratification by province, contributing veterinarian, and feedlot size.

Table 1a. Feedlot capacity (Number of cattle x 1000) of Saskatchewan and Alberta as of January 1, 2018. Source CANFAX, COF, accessed 12/31/2018

Province	Number of cattle (x1000)
Alberta	1333.3
Saskatchewan	90.5

Feedlot capacity information *per se* is not available for Ontario feedlots. However, this information is available from the CANFAX Statistical Briefer about Canadian fed cattle production in 2017:

Table 1b. Number of beef slaughter heifers and steers produced in 2017.

Province	Number of beef slaughter heifers and steers
Alberta	1142.8
Saskatchewan	148.3
Ontario	399.7
All 3 provinces	1690.8

From this information, could extrapolate that Ontario has 2.7x the feedlot capacity of Saskatchewan (399.7/148.3 = 2.7) but 0.35 the feedlot capacity of Alberta (399.7/1142.8 = 0.35). Therefore can extrapolate feedlot capacity for Ontario from this information by these numbers by values in Table 1a:
2.7 x 90.5 (x 1000) = 244.4 (x1000)
0.35 x 1333.3 = 466.7 (x1000)
Find average of these 2 estimates:
(244.4 + 466.7)/2 = 355.6 → Estimate of ON feedlot capacity = 355.6 x 1000

Table 1c. Extrapolation of feedlot capacity for Ontario and feedlot capacity for all 3 provinces based on Tables 1a and 1b.

Province	Number of cattle (x1000)	Percentage
Alberta	1333.3	74.9

Saskatchewan	90.5	5.1
Ontario	355.6	20.0
All 3 provinces	1779.4	100.0

Calculation of representative sample size of number of cattle for estimation of AMD treatment incidence performed with Epitools (http://epitools.ausvet.com.au) sample size calculator (for estimate of percentage with *a priori* estimate of proportion) based on expected treatment incidence (as performed in Timmerman *et al*, Quantification and evaluation of antimicrobial drug use in group treatments for fattening pigs in Belgium, *Preventive Veterinary Medicine* (2006) 74:251-263).

Used expected treatment incidence from Benedict *et al*, Antimicrobial Resistance in *Escherichia coli* Recovered from Feedlot Cattle and Associations with Antimicrobial Use, *PLOS One*, 2015. From Table 4: Pen level exposures to antimicrobial drugs. Sum of Average ADD per Animal-Day = 0.1183 ADD/animal-day → 118.3 ADD/1000 animals/day. Treatment incidence = 118.3/1000 = 0.12

Sample size to estimate a simple proportion (apparent prevalence)
Jan 23 05:55:53 2019

Inputs

Estimated Proportion	0.12
Desired precision of estimate	0.05
Confidence level	0.95
Population size	1779

Results

Sample size required for specified inputs

Population =	150

Recommended sample size is total of 150 x 1000 cattle stratified proportionately over three provinces of AB, ON, and SK.

Table 2. Recommended sample sizes to obtain representative sample of fed cattle in Alberta, Saskatchewan and Ontario

Province	Percentage	Recommended Sample Size (Number of cattle x 1000)
Alberta	74.9%	112.3
Saskatchewan	5.1%	7.7
Ontario	20.0%	30.0
All 3 provinces	100%	150

Table 3. Sampling Frame Available

Province	Number of feedlots	1000-5000	5001-10,000	10,001-20,000	20,001 and greater	Total estimated capacity number of cattle (x1000)
Alberta	79	23	26	19	11	877.3
Saskatchewan	3	0	0	2	1	52.0
Ontario	36	32	4	0	0	127.5
All 3 provinces	118	55	30	21	12	1056.8

Table 4. Alberta Feedlot Bunk Capacity as of January 1, 2018, information from CANFAX (http://www.canfax.ca/CattleOnFeed/BunkCapacity.aspx)

Bunk Capacity	Percentage of Cattle in Feedlots with this Bunk Capacity	Minimum Number of Cattle (x 1000) to be sampled
1000-5000	14.6%	16.4
5001-10,000	24.8%	27.9
10,001-20,000	25.6%	28.7
20,001 or more	35.0%	39.3
All	100%	112.3

- Based on the recommended sample sizes of cattle per province (Table 2), feedlots were randomly sampled (stratified by contributing veterinary clinic and feedlot size) from the sampling frame available (Table 3).
- In Alberta, since there is a wide range in feedlot sizes and it was desirable to proportionately represent the different management practices that might exist according to the size of the feedlot, an additional stratification step was added using information from CANFAX was to estimate the percentage of cattle that were in different categories of feedlot sizes in Alberta (1000-5000 cattle; 5001-10,000 cattle; 10,001 to 20,000 cattle; and 20,001 cattle and greater) (Table 4).
- Feedlots were randomly sampled until the number of cattle required per province (and in Alberta, per stratum of feedlot size) until the minimum number of cattle required per stratum was obtained AND each participating veterinary clinic that contributed a feedlot to the sampling frame was represented (Table 5)

Table 5. Total number of feedlots to be sampled by province and bunk capacity

Province	Number of feedlots	Bunk Capacity of Feedlot	

		1000-5000	5001-10,000	10,001 - 20,000	20,001 or greater	Available bunk capacity for sampling
Alberta	16	5	4	4	3	181.3
Saskatchewan	2	0	0	1	1	40
Ontario	8	6	2	0	0	33.5
All 3 Provinces	**26**	**11**	**7**	**5**	**3**	**254.3**

- In Alberta and Saskatchewan, the stipulation that all contributing veterinary clinics be represented in each stratum resulted in marked oversampling, i.e. many more cattle were selected than required (181.3 x 1000 cattle in Table 5 vs. 112.3 x 1000 required cattle in Table 2 for Alberta and 40.0 x 1000 cattle in Table 5 vs. 7.7 x 1000 required cattle in Table 2 for Saskatchewan).
- The number of cattle selected for sampling was roughly equal to the calculated required number of cattle in Ontario (33.5 x 1000 cattle in Table 5 vs. 30.0 x 1000 required cattle in Table 2), therefore the full one-time capacity of each feedlot will be sampled.
- If all randomly selected feedlots were fully sampled, the total number of sampled cattle would be 254.3 x 1000 cattle (Table 5) versus the recommended 150 x 1000 cattle (Table 2). Therefore, while the entire bunk capacity of feedlots will be sampled in Ontario feedlots since the recommended number of cattle vs. randomly selected cattle was similar, only partial bunk capacity of randomly selected feedlots will be sampled in Saskatchewan and Alberta.
- In Saskatchewan, two veterinary practices contributed cattle to the sampling frame. Since the required number of cattle for Saskatchewan is 7.7 x1000, closed production lots will be randomly sampled from the 2 feedlots selected for Saskatchewan until each feedlot contributes data from at least (7.7/2 = 3.85) or 3850 cattle.
- In Alberta, the number of cattle sampled must be corrected by requirement of cattle for the stratum and the contribution of each veterinary clinic to each stratum's sampling frame.
    - There are currently four veterinary clinics that have contributed cattle to the Alberta sampling frame and as previously mentioned there are four strata of feedlot bunk capacity size in Alberta.
    - The total number of cattle required for proportional representation of each stratum is shown in Table 4.
    - This number of cattle required for each stratum will be divided equally among the contributing veterinary clinics, and production lots will only be sampled from these feedlots until the required number of cattle is satisfied.
        - For example, in the 10,000 – 20,000 bunk capacity size bin, four feedlots representing each of the contributing veterinary clinics have been randomly selected because each of the veterinary clinics submitted feedlots for this stratum. If all of these feedlots had their bunk capacity fully sampled, this would result in sampling of about 60 x 1000 cattle, far exceeding the required number of cattle, which is 28.7 x 1000 cattle (Table 4).

    - 28.7 x 1000 divided by four is 7.2 x 1000, so production lots will be randomly selected from each feedlot until each feedlot contributes AMU data from approximately 7.2 x 1000 cattle resulting in collection of data for 28.7 x 1000 cattle from this bunk capacity bin.
  - In the >20,000 bunk capacity bin, only three of the veterinary clinics offered feedlots so three feedlots were randomly selected. If all three randomly selected feedlots were fully sampled, AMU data for 72.5 x 1000 cattle would be obtained while data is only required for 39.3 x 10000 cattle (Table 4).
    - 39.3 divided by three is 13.1 x 1000 cattle, so production lots will be randomly selected until each feedlot contributes data from 13.1 x 1000 cattle.
